# Stroke in Brazil: a neglected disease

**DOI:** 10.1590/S1516-31802005000100001

**Published:** 2005-01-02

The turning point in the transition from infectious diseases to non-transmittable diseases in Brazil occurred in the 1960s, taking the country as a whole.^1^ However, considering the main cities, such as São Paulo and Rio de Janeiro, cerebrovascular disease mortality rates have surpassed the *"old cardiovascular diseases"* like rheumatic heart disease and syphilitic aortic disease since the end of World War II.^2^ Until the end of the 1980s, the burden of stroke mortality observed in the main Brazilian cities was higher than in the United States, Canada and western European countries, and similar to what is observed in Eastern Europe and Japan.^3^ In 2002, the latest year with official health statistics available, considering all causes of deaths, stroke (either type) was the leading cause of mortality in Brazil with 87, 344 deaths, and coronary heart disease was the second most common cause with 81,505 deaths. This pattern is more evident for women (42,883 for stroke deaths and 34,563 for coronary heart disease deaths) than for men, and this is also the pattern for the poorest regions, such as the North (including the Amazon basin), Northeast, and Center-West.^4^

Although stroke is a major public health problem, there is little focus on the control of risk factors, organizing of medical care and funding of research into the field of cerebrovascular diseases. A search through PubMed [http://www.ncbi.nlm.nih.gov/entrez] using keywords like "stroke and Brazil" and "cerebrovascular and Brazil" yields few papers. Recently, a review of epidemiological surveys on stroke in South America^5^ highlighted only four case series in three Brazilian cities.^6-8^ The few papers published on stroke are at odds with Brazil’s general contribution to science, including the biomedical field.^9^

**Figure 1:**
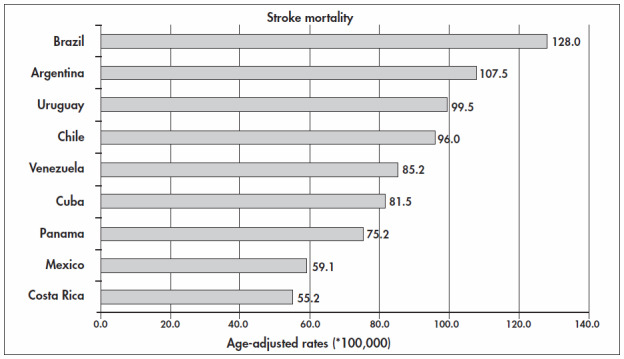
Age-adjusted stroke mortality rates among Latin American countries in 2002, for men of over 15 years old.^4,10^

**Figure 2:**
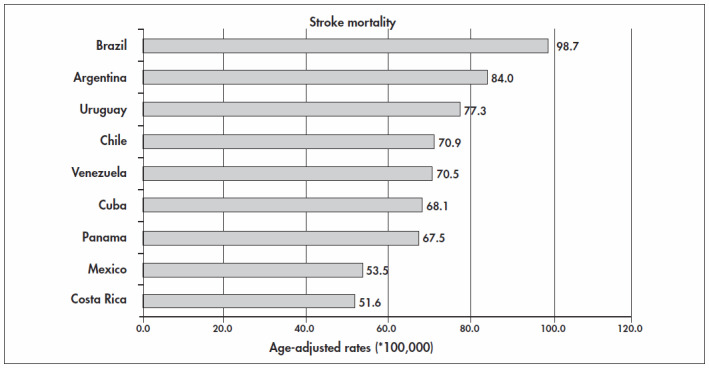
Age-adjusted stroke mortality rates among Latin American countries in 2002, for women of over 15 years old.^4,10^

To emphasize the importance of stroke in Brazil, we compared the mortality rates due to stroke in countries that have mortality system coverage of over 80%. Among Latin American countries, according to the mortality database that was obtained from the World Health Organization Statistical Information System (WHOSIS), downloaded on Aug 15, 2004, the countries that were eligible for such comparison were Argentina, Chile, Costa Rica, Cuba, Mexico, Panama, Venezuela and Uruguay. The mortality data for Brazil and its individual states was obtained from the files of the health statistics center of the Ministry of Health (http://www.datasus.gov.br). Figures 1 and 2 show that age-adjusted mortality rates for stroke in Brazil are the highest among the Latin American countries with good official health statistics. This is true for both men and women.^10^ The real picture is harsher than indicated by these rates because the data system coverage of all deaths in Brazil is the lowest among the countries in this comparison.

The higher burden of stroke mortality deaths in Brazil in comparison with other countries can be considered to be the consequence of social determinants, as shown in the unique population-based study performed by Lessa and coworkers in Salvador, Bahia, during the early 1980s.^11-13^ This revealed significant social determination of stroke distribution. Recently, new studies considering gender and the social exclusion index in São Paulo^14-16^ have shown that there is an important social determinant, and that this is the key to understanding why stroke is still a neglected disease in Brazil.
